# Chelidonine reduces IL-1β-induced inflammation and matrix catabolism in chondrocytes and attenuates cartilage degeneration and synovial inflammation in rats

**DOI:** 10.1590/1414-431X2023e12604

**Published:** 2023-08-14

**Authors:** Mao Li, Ying Zhu, Jiajia Shao, Chuanbing Wang, Bin Dong, Haiyong Cui, Dongdong Dai

**Affiliations:** 1 Department of Orthopaedics, The First Affiliated Hospital of Anhui University of Science and Technology (Huainan First People's Hospital), Huainan, Anhui, China; 2 Department of Stomatology, The First Affiliated Hospital of Anhui University of Science and Technology (Huainan First People's Hospital), Huainan, Anhui, China; 3 Department of Orthopaedics, Huainan Chaoyang Hospital, Huainan, Anhui, China; 4 Department of Orthopedics, Huainan Oriental Hospital Group Affiliated to Anhui University of Science and Technology, Huainan, Anhui, China

**Keywords:** Chelidonine, Inflammation, Osteoarthritis, NF-κB, Catabolism

## Abstract

Chondrocyte inflammation and catabolism are two major features in the progression of osteoarthritis (OA). Chelidonine, a principal alkaloid extracted from *Chelidonium majus*, is suggested to show anti-inflammation, anti-apoptosis, and anti-oxidation activities in various diseases. However, its potential effects on OA cartilage degeneration remains unclear. To evaluate the effect of chelidonine on OA and its underlying mechanism, we incubated chondrocytes with interleukin (IL)-1β and chelidonine at varying concentrations. Then, we performed the CCK-8 assay, fluorescence immunostaining, reverse transcription PCR, ELISA, and western blotting to evaluate cell viability, catabolic/inflammatory factors, levels of extracellular matrix (ECM)-related proteins, and the involved pathways. H&E and Safranin-O staining and ELISA were performed to measure cartilage degradation and synovial inflammation. Chelidonine suppressed the IL-1β-mediated catabolism and inflammation of chondrocytes. Chelidonine suppressed the NF-κB pathway activation. Similarly, our *in vivo* experiment showed that chelidonine partially attenuated cartilage degradation while inhibiting synovial inflammation. Chelidonine inhibited inflammation and catabolism through modulation of NF-κB pathways *in vitro*, thereby avoiding rat cartilage degeneration and synovial inflammation within OA.

## Introduction

Osteoarthritis (OA) has become a frequently occurring chronic degenerative arthropathy, affecting tens of millions of people around the world (1). OA is characterized by erosion of articular cartilage, articular hypertrophy and synovitis, as well as remodeling of subchondral bone (2), which can lead to serious arthralgia and dysfunction. At present, pharmaceutical drugs like bisphosphonates and non-steroidal anti-inflammatory drugs (NSAIDS) are used to alleviate symptoms but have serious adverse reactions. In this regard, drugs that delay OA development with high safety and effectiveness are urgently needed.

OA pathogenesis involves multiple factors, but cartilage destruction due to uncontrolled proteolytic destruction of the extracellular matrix (ECM) is certainly the most significant factor. Chondrocytes are the cells within articular cartilage that keep the synthesis-degradation balance of ECM and exert a vital function during OA progression (3). Inflammation has been suggested to induce chondrocyte catabolic activity, thereby resulting in ECM degradation (4). Interleukin (IL)-1β is one of the pro-inflammatory cytokines generated via the activation of catabolic activity in OA processes, which facilitates OA occurrence. IL-1β promotes the production of several proteolytic enzymes in chondrocytes, which include matrix metalloproteinases (MMPs) that function to increase chondrocyte catabolism through accelerating ECM degradation (5,6). In addition, aggrecan, an important component of the cartilage ECM, enhances the compression resistance of cartilage (7). Several studies have demonstrated that IL-1β is involved in degrading aggrecan (7). Such cleavage is possibly associated with some ADAMTS family members, including ADAMTS4 or ADAMTS5 (8). IL-1β stimulation greatly contributes to ADAMTS expression, thus promoting aggrecan degradation (7).

Chelidonine is an important alkaloid extracted from *Chelidonium majus*, a plant that has many pharmacological effects. Crude extracts of *C. majus* and its purified chelidonine exhibit wide bioactivities, like anti-inflammation, immunomodulation, analgesia, anti-biosis, and anti-virus (9,10). Chelidonine exhibits wide inhibitory activities in airway inflammation, which is achieved through suppressing eotaxin-2 and IL-4 (11). Chelidonine suppresses NF-κB pathways to mitigate inflammation induced by TNF-α within HCT116 cells (12). In addition, chelidonine inhibits TLR4/NF-κB pathway to suppress inflammatory factor production caused by lipopolysaccharide (LPS) within RAW264.7 macrophages (13). A previous study demonstrated that *C. majus* methanol extracts significantly suppressed the progression of collagen-induced arthritis and that this action was characterized by the decreased production of inflammatory cytokines, B cells (in spleen), and an increased proportion of CD4+CD25+ regulatory T cells *in vivo* (14). This study investigated the effect of *C. majus* methanol extract from the perspective of immunity. However, it also had some shortcomings, such as using a mixture, and the main component of the methanol extract of *C. majus* is not known. Furthermore, this study did not assess its effect on chondrocytes and synovial cells. Thus, the purpose of this study was to evaluate the effect of the main component chelidonine of *C. majus* on articular chondrocytes and synovial cells and its mechanism.

The present work examined the potential effects of chelidonine on interleukin (IL)-1β-mediated inflammatory response as well as the underlying mechanisms in chondrocytes. This work focused on investigating whether chelidonine was effective in treating OA-induced cartilage degeneration and synovial inflammation and the underlying mechanism through modulating the inflammation of chondrocytes *in vivo* and *in vitro*.

## Material and Methods

The present study was approved by the Animal Care and Use Committee of the Anhui University of Science and Technology (2020-LS-15). Each animal procedure was conducted in line with the Animal Management Rules of the Anhui University of Science and Technology (China).

### Isolation and culture of chondrocytes

Knee joint chondrocytes were collected from 4-week-old Sprague Dawley (SD) rats provided by Lab Animal Company (China). Articular cartilage was separated from rat knee joints, followed by digestion with 0.25% trypsin for a 1-h period at 37°C and then transferred onto 0.3% collagenase II under the same temperature for 6 h. Afterwards, we utilized a mesh screen for cell filtration. The cell suspension was transferred to the culture flask, cells were cultured with DMEM that contained 20% bovine serum albumin (BSA) and 1% penicillin-streptomycin and incubated at 37°C and 5% CO_2_ conditions. The second passage of cells was used in later analyses.

### Cell viability assay

A CCK-8 assay kit (Beyotime, China) was used to measure cell viability according to specific instructions. Briefly, cells reaching confluence (1×10^5^/well) were cultured in 96-well plates for 24 h. Afterwards, cells were treated or not with different concentrations of chelidonine (2.5-100 μM), with or without IL-1β (10 ng/mL). After 12 h, CCK-8 solution (10 μL) was added to cells followed by incubation for 2 h. Later, the microplate reader (iMARK, Bio-Rad, USA) was employed to measure the absorbance at 450 nm.

### Western blotting assay

We isolated total cellular proteins and lysed them using RIPA lysis buffer that contained phenylmethanesulfonyl fluoride (1 mM). Thereafter, each sample was subject to 30 min of on-ice incubation. Later, we centrifuged cells for 15 min at 14,000 *g* and 4°C and determined protein contents by adopting the BCA protein detection kit. Then, 40 ng of protein was separated on the SDS-PAGE and transferred onto the PVDF membranes (BioRad, US). Subsequently, 5% skimmed milk was utilized to block membranes under ambient temperature for a 2-h period, followed by incubation using the anti-p65 and anti-β-actin primary antibodies under 4°C overnight and further incubation using corresponding secondary antibodies under ambient temperature for 2 h. The electrochemiluminescence (ECL) PLUS reagent (Invitrogen, USA) was used for blot visualization. The Image J 3.0 software (BioRad) was employed for intensity quantification.

### Enzyme-linked immunosorbent assay (ELISA)

Using the monoclonal antibody-based mouse IL ELISA kit, ELISA was conducted to measure ILs (including IL-6, IL-12, IL-1β, TNF-α, and IFN-γ) in line with specific protocols (R&D Systems, USA). Results are reported as means±SE from three or more individual assays and analyzed by ANOVA.

### qRT-PCR assay

Using the RNA extraction kit (Thermo Fisher Scientific, USA), we isolated total cellular RNAs from chondrocytes. Thereafter, we quantified the extracted total cellular RNA through spectrophotometry and prepared cDNA using the RT-First Strand cDNA Synthesis kit (Thermo, K1622) through reverse transcription in line with specific protocols. Using the PCR Master Mix Kit (Promega, USA), the qRT-PCR procedure was carried out using the CFX96TM Real-Time PCR system (BioRad). The 2^-ΔΔct^ approach was utilized for data analysis, with β-actin being the endogenous reference. The primers of targeted genes are listed in Table 1.

**Table 1:** Primer sequences of targeted genes.

Genes	Primer sequences
MMP-1	5′-GCTTAGCCTTCCTTTGCTGTTGC-3′ (forward)
	5′-GACGTCTTCACCCAAGTTGTAGTAG-3′ (reverse)
MMP-3	5′-CTGGGCTATCCGAGGTCATG-3′ (forward)
	5′-TGGACGGTTTCAGGGAGGC-3′ (reverse)
MMP-13	5′-AGCCACTTTATGCTTCCTGATG-3′ (forward)
	5′-GATGTTTAGGGTTGGGGTCTTC-3′ (reverse)
ADAMTS-4	5′-AAGCATCCGAAACCCTGTCAACG-3′ (forward)
	5′-AGCCATACCCAGAGCGTCAC-3′ (reverse)
ADAMTS-5	5′-AGAGTCCGAACGAGTTTACG-3′ (forward)
	5′-GTGCCAGTTCTGTGCGTC-3′ (reverse)
Aggrecan	5′-GTCAGATACCCCATCCACACTC-3′ (forward)
	5′-CATAAAAGACCTCACCCTCCAT-3′ (reverse)
Collagen II	5′-CTCAAGTCGCTGAACAACCA-3′ (forward)
	5′-GTCTCCGCTCTTCCACTCTG-3′ (reverse)
β-actin	5′-TCAGGTCATCACTATCGGCAAT-3′ (forward)
	5′-AAAGAAAGGGTGTAAAACGCA-3′ (reverse)

### Immunofluorescence staining

Chondrocytes (1×10^5^/mL) were implanted onto 6-well plates with p65 staining and later classified into 3 groups, including control, IL-1β, and IL-1β+Cheli groups in which cells were treated with 10 ng/mL IL-1β and IL-1β + Cheli for 24 h. Afterwards, cells were incubated for 10 min at room temperature for 4% paraformaldehyde fixation. After washing with PBS thrice, cells were further exposed to 0.1% Triton x-100 treatment for 15 min. Then, 10% goat serum was utilized to block cells for 30 min, followed by overnight incubation using anti-p65 primary antibody in a box under 4°C. On day 2, the specimens were incubated with secondary antibody for 1 h under 37°C. At last, DAPI was utilized to stain the cell nuclei. Later, we chose 5 fields of view at random for observation under the fluorescence microscope (Olympus Inc., Japan). An observer blinded to experimental grouping was invited to measure fluorescence intensity with the ImageJ software (NIH, USA).

### OA animal model

Male SD rats (200-250 g) were raised in the laboratory under standard conditions (20-24°C, 50-55% humidity, 12-h light/dark cycle). All animals were allowed to eat food and drink water freely. Then, they were randomized into 3 groups, including sham, OA, and OA+Cheli groups (n=3). Each animal was given an intraperitoneal injection with 50 mg/kg pentobarbital sodium for anesthesia. To construct the OA rat model, the anterior cruciate ligament was transfected, and then the medial menisci (ACLT+MMx) in right knee was resected, according to a previous description (15). After rinsing with sterile saline, the skin and capsule were sutured. The OA+chelidonine group was orally administered 5 mg/kg chelidonine daily (16). Rats in the other groups received equal volumes of saline. At 8 weeks postoperatively, each animal was killed for further experiments. Each group was raised in one cage. Eight weeks after the operation, all rats were euthanized by CO_2_ inhalation (40% vol/min for 5 min), and relevant joint fluid and tissue samples were collected for further experiments.

### Histological analysis

After surgical resection, the right knee joints were placed into 4% (v/v) paraformaldehyde for a 48-h fixation. Cartilage tissues were later de-calcified with Calci-Clear slow solution (Beyotime Biotechnology, China) that contained 10% (w/v) EDTA (pH 7.4) for around 3 weeks, followed by paraffin embedding. Cartilage tissues were then cut into sections, which were later stained with hematoxylin and eosin (H&E) and safranin-O to evaluate cartilage degeneration under a light microscope (Olympus, China). The degree of cartilage degeneration was assessed by the Osteoarthritis Research Society International (OARSI) scores (17), and the observer was blinded to the treatment protocol.

### Immunohistochemical analysis

The paraffin sections (5 μm) were deparaffinized in xylene, rehydrated in ethanol, and blocked with 3% (v/v) H_2_O_2_ for 10 min followed by treatment in 5% BSA for 30 min and then incubated overnight with primary antibodies (anti-CD68). The slides were treated by a secondary antibody for 60 min and counterstained with hematoxylin for 8 min. Images were captured with a light microscope. The specimen was assessed by counting the CD68-positive cells in 10 different regions of the synovium.

### Statistical analysis

Each experiment was carried out at least three times. Results are reported as means±SD. The variance between the experimental groups was estimated using one-way ANOVA and Tukey's *post hoc* test. P<0.05 was regarded as statistical significance.

## Results

### Effects of chelidonine on chondrocyte cell viability

**Figure 1:**
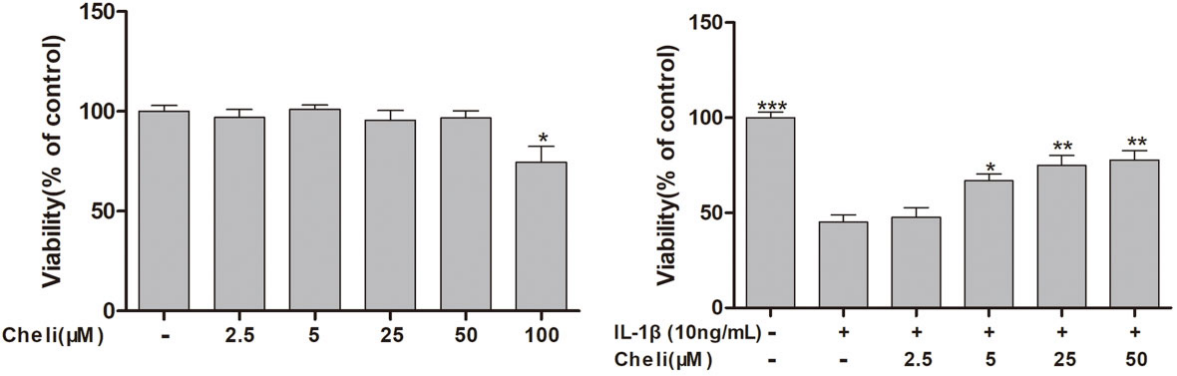
Role of chelidonine (Cheli) in rat chondrocyte viability. **A**, Cytotoxicity of chelidonine at varying doses for 12 h to chondrocytes measured by the CCK-8 method. **B**, CCK-8 analysis of chelidonine-treated chondrocytes stimulated by IL-1β. Data are reported as means±SD. *P<0.05, **P<0.01, ***P<0.001 compared to IL-1β group (ANOVA).

For predicting cytotoxicity of chelidonine in chondrocytes, chelidonine at varying doses (0, 2.5, 5, 25, 50, and 100 μM) was used to treat cells for 12 h. The results showed that chelidonine at 0-50 μM did not exert any cytotoxicity to chondrocytes (Figure 1A), while treatment with chelidonine at >5 μM attenuated the IL-1β-induced chondrocyte apoptosis (Figure 1B). Therefore, chelidonine at 5 and 25 μM were selected for later analysis.

### Chelidonine inhibited the IL-1β-induced production of inflammatory cytokines in primary chondrocytes

**Figure 2:**
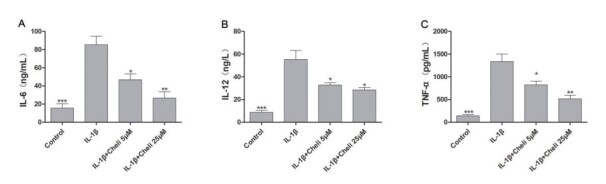
. Role of chelidonine (Cheli) in interleukin (IL)-1β-mediated chondrocyte inflammation. **A**, Chelidonine inhibited IL-1β-mediated expression of inflammation-associated proteins in a concentration dependent manner. Levels of IL-6 (**A**), IL-12 (**B**), and tumor necrosis factor (TNF)-α (**C**) proteins were analyzed through ELISA. Data are reported as means±SD. *P<0.05, **P<0.05, ***P<0.05 compared to IL-1β group (ANOVA).

Inflammatory factor levels in the supernatant of chondrocyte culture were measured through ELISA. IL-1β treatment significantly increased IL-6, IL-12, and TNF-α levels compared to the control group (Figure 2). In addition, the results suggested that chelidonine suppressed the IL-1β-mediated increased inflammatory factor expression in a dose-dependent manner, indicating that chelidonine exerted anti-inflammatory effects on IL-1β-induced chondrocytes.

### Role of chelidonine in IL-1β-induced NF-κB pathway activation in chondrocytes

**Figure 3:**
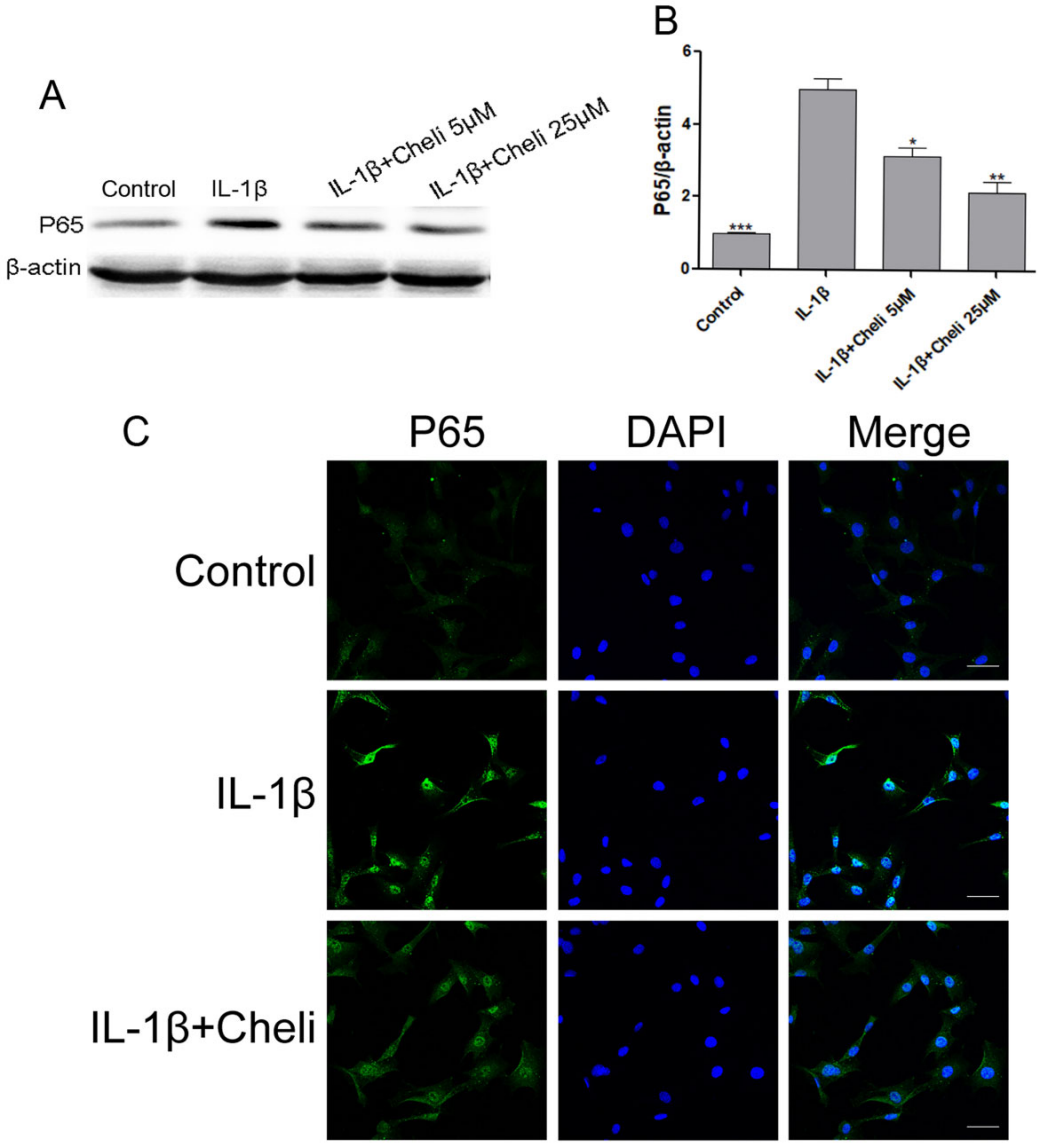
Role of chelidonine (Cheli) in interleukin (IL)-1β-mediated NF-κB pathway activation in chondrocytes. **A** and **B**, p65 expression was analyzed through western blot assay. **C**, p65 transnuclear level was measured through DAPI and immunofluorescence staining (scale bar: 50 µm). Data are reported as means±SD. *P<0.05, **P<0.05, ***P<0.05 compared to IL-1β group (ANOVA).

As shown in Figure 3A and B, IL-1β significantly promoted NF-κB p65 expression in chondrocytes, while chelidonine dose-independently suppressed the IL-1β-induced NF-κB p65 expression. Immunofluorescence of chondrocytes responsive to IL-1β-mediated NF-κB activation was measured to explore the role of chelidonine in the nuclear translocation of NF-κB p65. Clear and enhanced nuclear p65 staining was observed in chondrocytes upon IL-1β stimulation, indicating the nuclear translocation of NF-κB p65, whereas chelidonine reversed the translocation of the NF-κB p65 subunit (Figure 3C). These findings may indicate that chelidonine exerted protective effects on chondrocytes induced by IL-1β by the NF-κB pathway.

### Chelidonine decreased catabolism in chondrocytes

**Figure 4:**
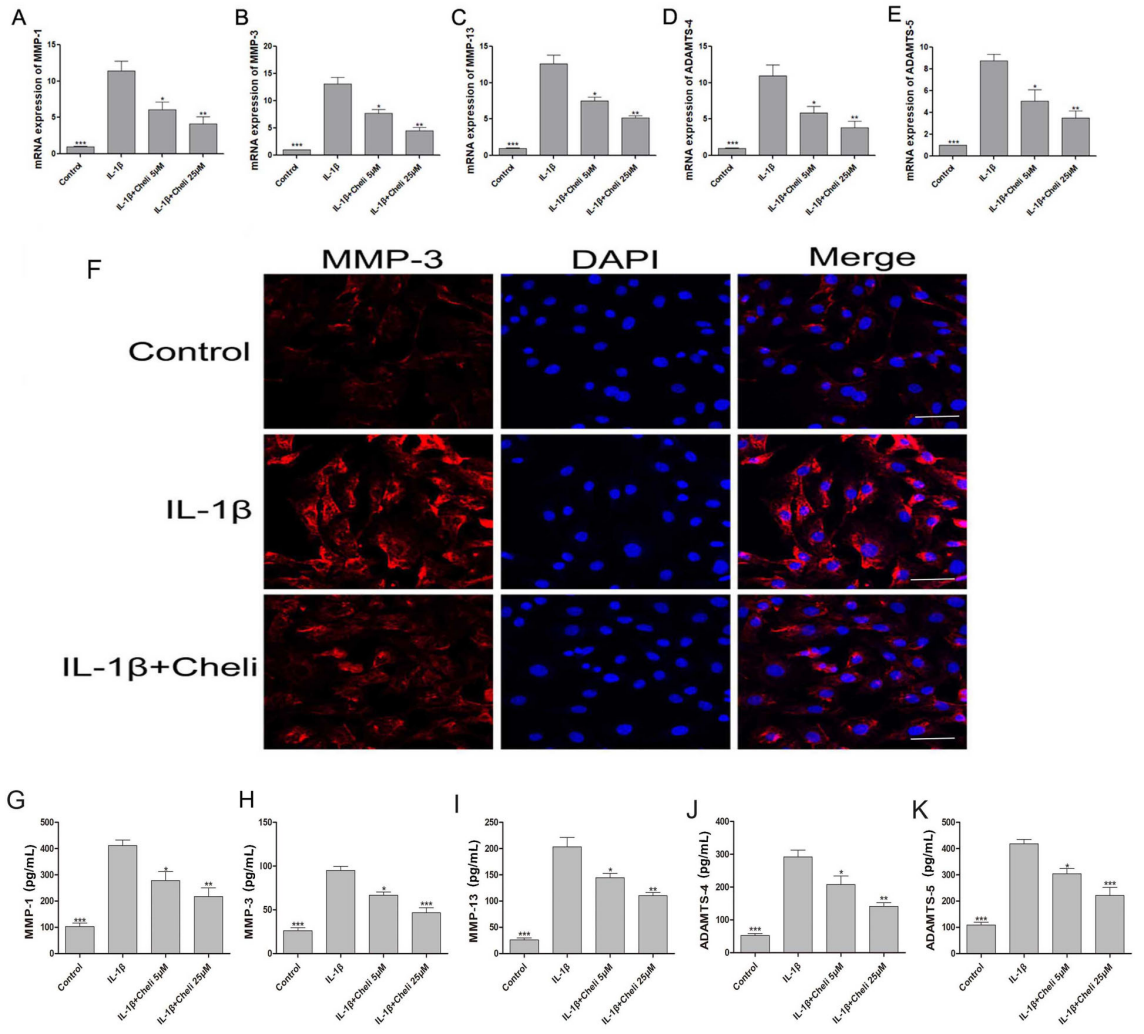
Effects of chelidonine (Cheli) on interleukin (IL)-1β-induced catabolism in chondrocytes. **A**, Chelidonine dose-dependently suppressed IL-1β-induced expression of catabolism-related marker genes. The mRNA expression levels of MMP-1 (**A**), MMP-3 (**B**), MMP-13 (**C**), ADAMTS-4 (**D**), and ADAMTS-5 (**E**) were measured by qPCR. **F**, Immunofluorescence evaluation of MMP3 protein in chondrocytes (scale bar: 50 µm). Levels of MMP-1 (**G**), MMP-3 (**H**), MMP-13 (**I**), ADAMTS-4 (**J**), and ADAMTS-5 (**K**) proteins were analyzed through ELISA. The results are reported as means±SD. *P<0.05, **P<0.05, ***P<0.05 compared to IL-1β group (ANOVA).

Since catabolic enzymes, including MMPs and ADAMTS, have important functions during OA progression, we explored the ability of chelidonine to affect the production of catabolic factors MMP1, MMP3, MMP13, ADAMTS4, and ADAMTS5, which play essential roles in cartilage degeneration. These results revealed that chelidonine decreased the release of MMP1, MMP3, MMP13, ADAMTS4, and ADAMTS5 in the IL-1β-stimulated group, decreasing catabolic activities in chondrocytes (Figure 4A-E). ELISA results and immunofluorescence evaluation of MMP3 protein expression was consistent with the mRNA results (Figure 4F-K), indicating chelidonine may regulate the ECM metabolic activity in chondrocytes.

### Chelidonine promoted anabolism in IL-1β-induced chondrocytes

**Figure 5:**
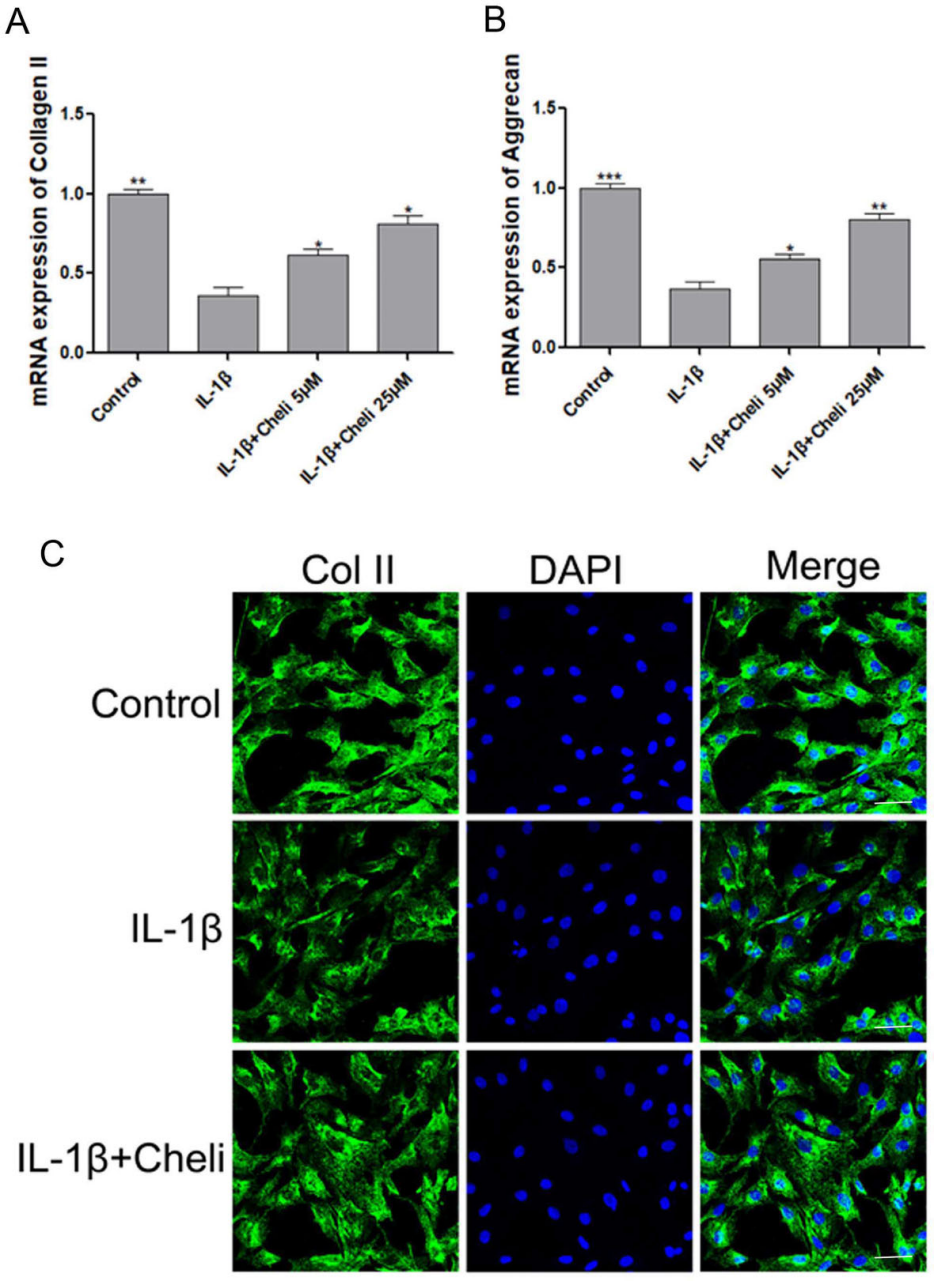
Effects of chelidonine (Cheli) on interleukin (IL)-1β-induced anabolism in chondrocytes. **A** and **B**, The relative mRNA expression of collagen-II and aggrecan in each group relative to Control. **C**, Immunofluorescence evaluation of collagen-II protein in chondrocytes (scale bar: 50 µm). The results are reported as means±SD. *P<0.05, **P<0.05, ***P<0.05 compared to IL-1β group (ANOVA).

We detected the major matrix proteins aggrecan and type II collagen in chondrocytes by PCR. Our results showed that chelidonine attenuated the IL-1β-mediated reduction of ECM components (aggrecan and type II collagen) (Figure 5A and B). Immunofluorescence evaluation of type II collagen protein expression was consistent with the mRNA results (Figure 5C). Together, all the above results demonstrated that chelidonine promoted anabolism in IL-1β-treated chondrocytes.

### Chelidonine inhibited synovial inflammation and protected cartilage in surgery-induced OA in rats

**Figure 6:**
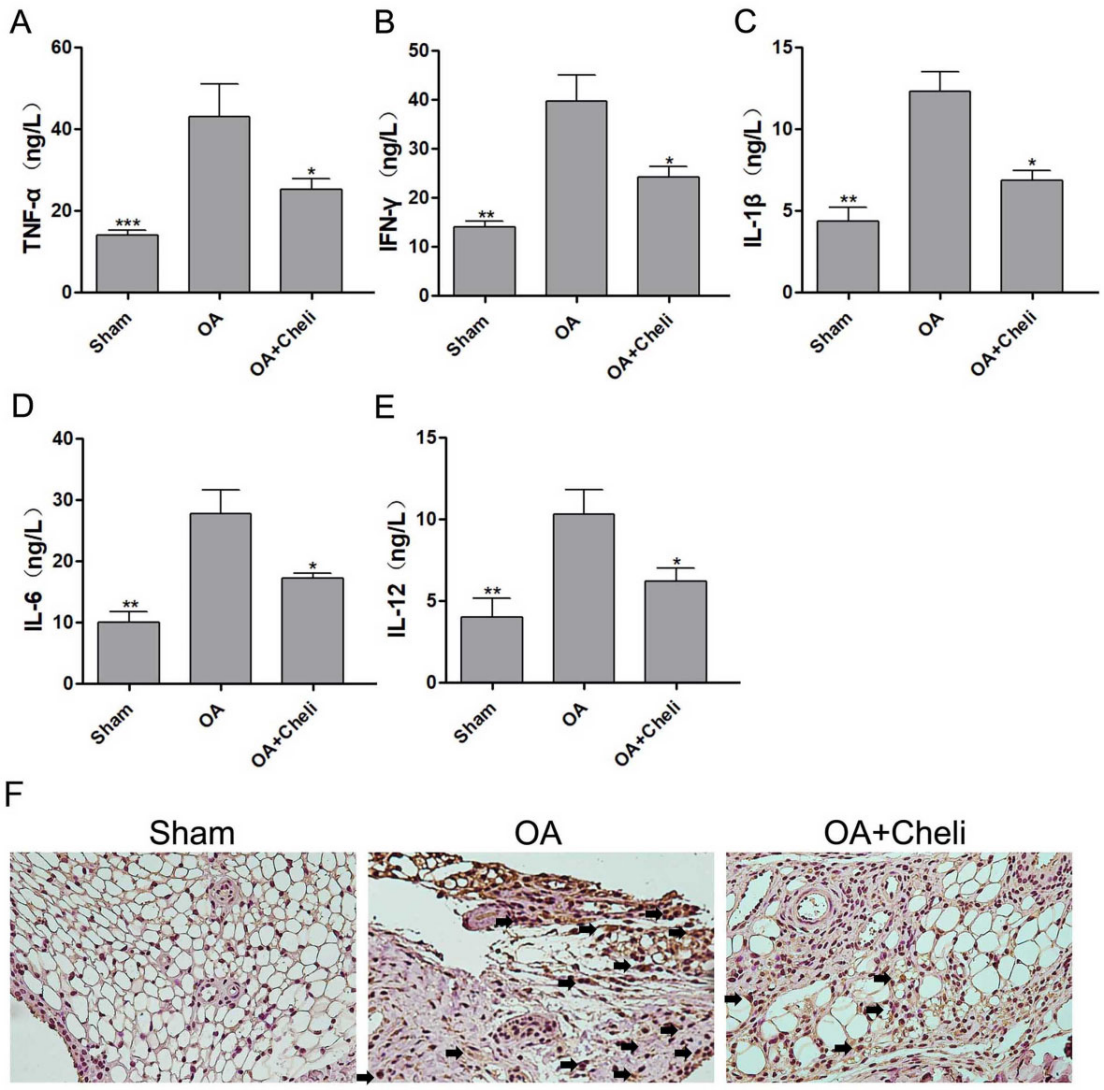
Effects of chelidonine on synovial inflammation in osteoarthritis (OA) rats. **A**-**E**, Chelidonine suppressed synovial inflammation in OA rats. The protein expression levels of tumor necrosis factor (TNF)-α (**A**), interferon (IFN)-γ (**B**), interleukin (IL)-1β (**C**), IL-6 (**D**), and IL-12 (**E**) were measured by ELISA. **F**, Representative synovial tissues stained with CD68 (40×, scale bar 100 μM), arrows indicate positive cells). The results are reported as means±SD. *P<0.05, **P<0.05, ***P<0.05 compared to the OA group (ANOVA).

In order to better examine chelidonine's protection of cartilage, the effect of chelidonine was evaluated by the OA rat model, and synovitis factors were measured through ELISA. Surgery increased the levels of IL-6, IL-12, IL-1β, IFN-γ, and TNF-α in knee synovial fluid, while chelidonine treatment had opposite effects (Figure 6). Immunohistochemical results showed that there were more CD68-positive cells in the OA group than in the sham group. Furthermore, chelidonine reduced the number of CD68-labeled inflammatory cells. All results demonstrated that chelidonine inhibited synovial inflammation in OA rats.

### Effects of chelidonine on OA cartilage histopathology

**Figure 7:**
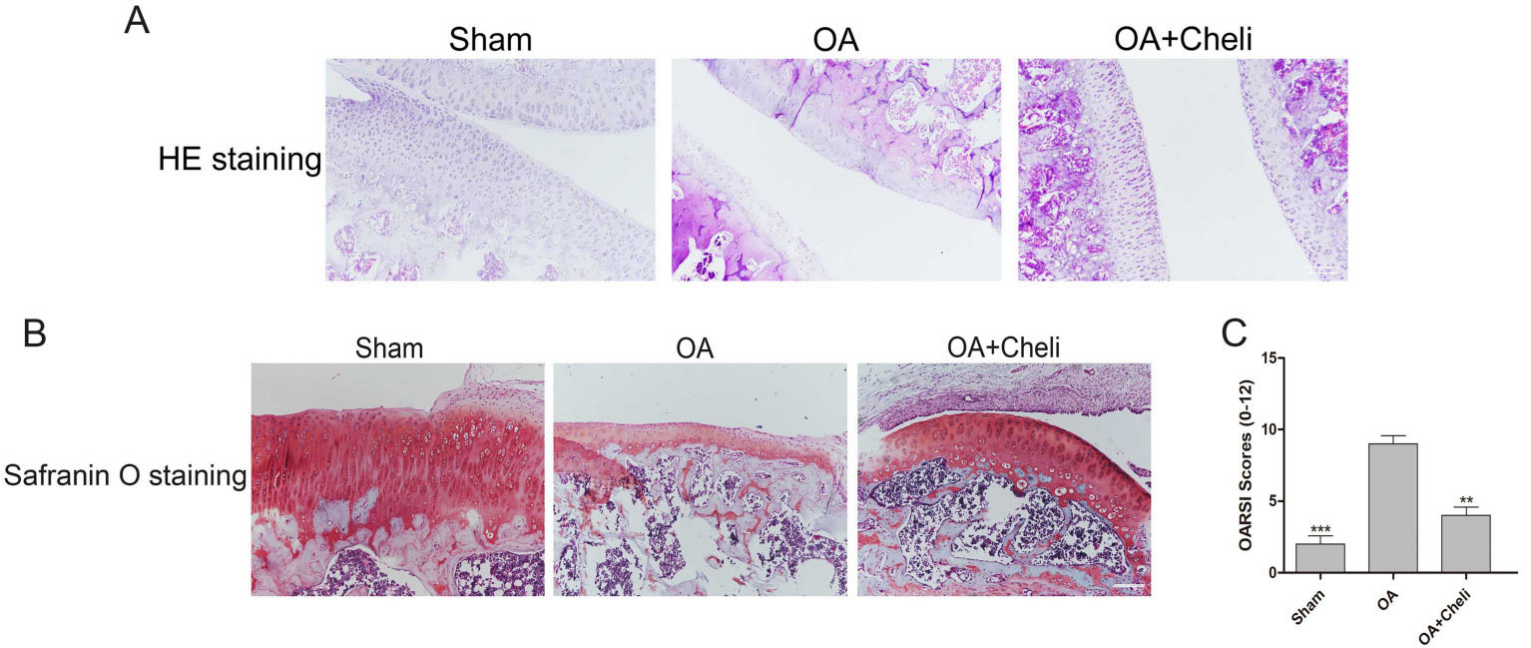
Gross morphology and histology of articular cartilages in rats. **A**, Hematoxylin and eosin (HE) staining of each group at 6 weeks (10×, scale bar 200 μM). **B**, Safranin-O staining of each group at 6 weeks (10×, scale bar 200 μM). **C**, Osteoarthritis Research Society International (OARSI) scores of each group at 6 weeks. The results are reported as means±SD. **P<0.05, ***P<0.05 compared to the osteoarthritis (OA) group (ANOVA).

We evaluated the morphologic and histopathologic changes in the cartilage surface and matrix layer (Figure 7). Compared with the sham-operation group, the OA-induction group suffered severe articular cartilage destruction. Furthermore, chelidonine treatment significantly increased the thickness of knee articular cartilage and mitigated cartilage degeneration. Compared with the OA group, the OA+chelidonine group had less severe cartilage degeneration, which was consistent with OARSI scores. Chelidonine protected the cartilage and significantly enhanced OA cartilage recovery.

## Discussion

Firstly, in this study, we reported the effect of chelidonine on the expression, production, and protein degradation activity of MMPs *in vitro* in IL-1β-induced chondrocytes as well as cartilage degradation and synovial inflammation *in vivo* in the OA rat model. *In vitro*, chelidonine suppressed the IL-1β-mediated catabolism (MMP1, MMP3, MMP13, ADAMTS4, and ADAMTS5) and inflammatory cytokines (IL-6, IL-12, and TNF-α) of chondrocytes. Chelidonine suppressed the NF-κB pathway activation. Similarly, our *in vivo* experiment showed that chelidonine partially attenuated cartilage degradation, while inhibiting synovial inflammation (IL-6, IL-12, IL-1β, IFN-γ, and TNF-α) and decreasing CD68-labeled inflammatory cells.

At present, the treatment and repair of OA-related cartilage degeneration is still a challenge that deserves more study. In early-to-mid OA stages, agents that promote cartilage repair have been widely applied clinically. ECM has a key function in keeping chondrocyte structure and function, while severe ECM degradation is observed in OA patients (18). The widely applied agents, including chondroitin sulfate, glucosamine polysaccharides, and hyaluronic acid, exert their effect by enhancing cartilage ECM formation while delaying collagen framework decomposition, thereby forming a molecular barrier and suppressing joint local inflammatory response for protecting chondrocyte activity (19,20). Aggrecan and collagen II are the main ECM components, while IL-1β can promote their degradation; therefore, agents reversing such degradation are promising in the treatment of OA (21,22).

OA represents one of the chronic disorders affecting elderly people, which is usually accompanied by chronic inflammation. Pro-inflammatory factors like IL-1β and TNF-α have important functions during OA development (23). ECM mostly contains collagens, which will be decomposed via MMPs. In cartilage tissues of OA cases, collagen levels decrease, while MMPs levels increase (24). It has been previously suggested that IL-1β triggers MMPs transcription in rabbit chondrocytes by increasing the serum amyloid A, one of the pro-inflammatory proteins (25). Additionally, pro-inflammatory factors also cause cartilage decomposition by activating MMPs (5). Additionally, IL-1β up-regulates pro-inflammatory gene levels, such as MMP-1, -3, -9, and -13, while inhibiting aggrecan production (26). MMPs are the proteolytic enzymes responsible for degrading ECM components within OA (27). Among them, MMP-13 has been suggested to be the pivotal factor in cartilage decomposition, due to its effect on collagen II cleavage (28). Cartilage ECM component decomposition is the OA hallmark (29,30). ADAMTSs, in particular ADAMTS-4 and ADAMTS-5, have important effects on regulating OA decomposition. IL-1β has been suggested to enhance ADAMTS-4 and ADAMTS-5 generation, thereby accelerating aggrecan decomposition in an indirect manner (31). Our findings indicated the role of chelidonine in preventing IL-1β-mediated ECM catabolism in chondrocytes. Furthermore, our *in vivo* study showed that chelidonine treatment increased the thickness of cartilage and mitigated cartilage degradation. Based on the above interesting results, we explored the mechanism by which chelidonine protects chondrocytes.

Pro-inflammatory cytokines including IL-1β and TNF-α have important functions during OA development (23). They can also increase gene transcription levels of TNF receptor 1, Fas, and Fas ligand, thereby promoting the mitochondrial production of cytochrome c and increasing chondrocyte apoptosis through the activation of caspase genes (32). In addition, such inflammatory factors can combine with receptors on the articular cartilage surface to activate the NF-κB, p38 MAPK, JNK, and ERK1/2 pathways in chondrocytes. They can also induce MMPs production for proteoglycan and collagen degradation in cartilage (33-35). The NF-κB pathway accounts for an important regulating factor for chondrocyte inflammation (36,37), and its activation plays a key role in producing inflammatory factors like iNOS and COX-2 (38). NF-κB deactivation is reported to significantly suppress the levels of MMP-3 and MMP-13 (39), and many articles suggest that the suppression of NF-κB pathway reduces the degradation of collagen II and aggrecan, thus postponing OA development. Together, this work suggested that chelidonine inhibited the IL-1β-mediated inflammation by suppressing NF-κB pathway to some extent. The interaction between chelidonine and OA needs further study.

### Conclusion

To sum up, this work demonstrated the effect of chelidonine in suppressing IL-1β-mediated inflammation and the related catabolism *in vitro*, and in protecting against OA in the rat model *in vivo*. The primary mechanism of chelidonine was related to the inhibition of NF-κB signaling. Therefore, our results indicated the potential of chelidonine in the treatment of OA.
